# Time-Resolved Systems Medicine Reveals Viral Infection-Modulating Host Targets

**DOI:** 10.1089/sysm.2018.0013

**Published:** 2019-03-28

**Authors:** Christian Wiwie, Irina Kuznetsova, Ahmed Mostafa, Alexander Rauch, Anders Haakonsson, Inigo Barrio-Hernandez, Blagoy Blagoev, Susanne Mandrup, Harald H.H.W. Schmidt, Stephan Pleschka, Richard Röttger, Jan Baumbach

**Affiliations:** ^1^ Institute of Mathematics and Computer Science, University of Southern Denmark, Odense, Denmark.; ^2^ Institute of Medical Virology, Justus Liebig University Giessen, Giessen, Germany.; ^3^ Center of Scientific Excellence for Influenza Viruses, National Research Centre, Cairo, Egypt.; ^4^ Department of Biochemistry and Molecular Biology, University of Southern Denmark, Odense, Denmark.; ^5^ Department of Pharmacology and Personalized Medicine, Maastricht Centre for Systems Biology (MaCSBio), Maastricht University, Maastricht, the Netherlands.; ^6^ Department of Experimental Bioinformatics, Technical University of Munich, Freising, Germany.

**Keywords:** drug discovery, influenza A, time series clustering, network enrichment, network pharmacology, host factors

## Abstract

**Introduction:** Drug-resistant infections are becoming increasingly frequent worldwide, causing hundreds of thousands of deaths annually. This is partly due to the very limited set of protein drug targets known for human-infecting viral genomes. The eleven influenza virus proteins, for instance, exploit host cell factors for replication and suppression of the antiviral immune responses. A systems medicine approach to identify relevant and druggable host factors would dramatically expand therapeutic options. Therapeutic target identification, however, has hitherto relied on static molecular networks, whereas in reality the interactome, in particular during an infection, is subject to constant change.

**Methods:** We developed time-course network enrichment (TiCoNE), an expert-centered approach for discovering temporal response pathways. In the first stage of TiCoNE, time-series expression data is clustered in a human-augmented manner to identify groups of biological entities with coherent temporal responses. Throughout this process, the expert can add, remove, merge, or split temporal patterns. The resulting groups can then be mapped to an interaction network to identify enriched pathways and to analyze cross-talk enrichments and depletions between groups. Finally, temporal response groups of two experiments can be intersected, to identify condition-variant response patterns that represent promising drug-target candidates.

**Results:** We applied TiCoNE to human gene expression data for influenza A virus infection and rhino virus infection, respectively. We then identified coherent temporal response patterns and employed our cross-talk analysis to establish two potential timelines of systems-level host responses for either infection. Next, we compared the two phenotypes and unraveled condition-variant temporal groups interacting on a networks level. The highest-ranking ones we then validated via literature search and wet-lab experiments. This not only confirmed many of our candidates as previously known, but we also identified phospholipid scramblase 1 (encoded by *PLSCR1*) as a previously not recognized host factor that is essential for influenza A virus infection.

**Conclusion:** With TiCoNE we developed a novel approach for conjointly analyzing molecular networks with time-series expression data and demonstrated its power by identifying temporal drug-targets. We provide proof-of-concept that not only novel targets can be identified using our approach, but also that anti-infective drug target discovery can be enhanced by investigating temporal molecular networks of the host in response to viral infection.

## Introduction

Drug discovery, in particular for anti-infective therapies, is in a deep crisis due to low and further declining efficacy in principle^1^ and drug resistance by constant adaptation of the pathogen by mutation, in particular.^2,3^ Systems medicine (also known as network medicine) proposes a more holistic approach, in this case by including also the host interactome to define essential host factors for the pathogen and, therefore, represents a potential solution to this roadblock.^4^ However, even this innovative approach relies on static networks, whereas in reality the underlying interactome, in particular during an infection,^5^ is subject to constant changes, facilitating its controllability by the cell.^6^

Traditional gene expression and network-based target identification has so far provided only few validated therapeutic approaches^6–8^ and mostly *in silico* predictions only.^9^ We hypothesize that this may be since all hitherto approaches were based on static networks, whereas temporal networks have a fundamental advantage in controllability and possibly also for drug discovery.^6^

Such network temporality is often modeled over time through dynamic links that can be active or nonactive (binary). We hypothesize that it is better modeled quantitatively and based on the temporal expressions profiles of the nodes (i.e., node dynamics rather than edge dynamics). Big “omics” data sets, including an increasing number of time-resolved data sets, as well as molecular networks, have been curated already. However, suitable approaches to analyze these data types together are lacking. Thus, an integrated analysis to illuminate systemic response patterns of temporal resolution has been infeasible so far.

**FIG. 1.**
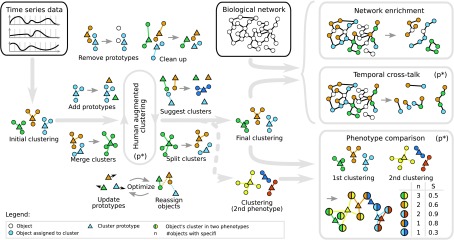
Principle of TiCoNE. TiCoNE is a novel temporal systems medicine tool for drug target discovery, network enrichment, and subnetwork cross talk detection over time. After an initial clustering of a given set of temporal expression profiles, the partitioning is continuously refined through human-augmented operations. We call this novel strategy “human-augmented clustering.” Clusters are represented by prototypes (triangles), and objects (circles) are assigned to the most similar cluster prototype. Given an interaction network of biological entities such as genes (circles), TiCoNE may further unravel subnetworks significantly enriched with disease genes (*de novo* network enrichment), compare these temporal network modules between different phenotypes, and identify enrichment or depletion of interactions (cross talk) between such temporal modules (temporal cross talk). We developed tailored statistical models to assess enrichment significance at different steps of data analysis (p*). TiCoNE, time course network enrichment.

We developed time course network enrichment (TiCoNE), a novel human-augmented time series clustering method combined with a temporal network enricher (Fig. 1) that enables drug target discovery based on temporal networks. Temporal gene clusters are embedded into molecular networks, and TiCoNE identifies molecular pathways (subnetworks) with a differential behavior under two conditions (e.g., diseases). Such temporal disease pathway candidates are evaluated by calculating empirical *p*-values (see Computational Methods section in Supplementary Data).

TiCoNE works with most kinds of biological entities (genes, proteins, RNAs, etc.) and most types of molecular measures acquirable for them (transcriptomics, proteomics, etc.). To increase readability, in the remainder of this article we simply refer to genes and gene expression as we focus on applying TiCoNE to influenza A virus (IAV) transcriptomics data.

## Materials and Methods

In this study, we briefly outline the computational approach of TiCoNE and the utilized data sets. For an exhausting and more formal description of the methodology, we refer to Computational Methods section in Supplementary Data. Details on the wet laboratory analysis can be found in Wet Laboratory Validation section in Supplementary Data.

### Overview

TiCoNE is a human-augmented clustering method for time series data combined with a temporal network enricher. As any clustering approach it seeks to partition objects of a data set into groups such that (1) objects of the same group are similar and (2) objects of different groups are dissimilar to each other. In this study, we group objects based on their time course. Since TiCoNE is human augmented, it allows the user to interfere with the clustering process and, thus, can incorporate valuable domain knowledge. After the clustering process, TiCoNE offers a sophisticated set of methods for an enrichment of the clustering with molecular networks paving the way for unprecedented systems medicine analyses of time series data.

### Clustering

The objective of clustering is to cluster biomolecular entities (e.g., genes) in such a way that the time courses of genes assigned to one cluster are more similar to each other than to time courses of genes assigned to different clusters. To establish the similarity between two time courses, the user can choose between the Euclidean distance and the Pearson correlation coefficient, depending on whether one wants to emphasize the time course amplitudes or their shapes, respectively. Note that TiCoNE can also directly cope with (biological or technical) replicates.

With this information and some optional data cleaning (e.g., removing genes with baseline time courses behavior), TiCoNE produces an initial clustering using one of several common clustering approaches. One may choose between CLARA,^10^ k-means,^11^ PAMK,^12^ STEM,^13^ and transitivity clustering.^14^ Once the initial clustering is identified, TiCoNE applies a prototype-based clustering scheme^15,16^ and behaves similar to k-means^11^: Each cluster is represented by a prototype and the following two steps are performed alternatingly:

1. Assign all genes (based on their time courses) to the most similar prototype.
2. Update the prototypes accordingly.

This process can be repeated automatically until convergence (i.e., no genes reassigned to a different cluster/prototype). The essential working mode of TiCoNE, however, is to allow the user to interfere with the clustering after each iteration in various ways. We term this approach human-augmented clustering.

### Human-augmented clustering

The result of each clustering iteration is presented to the user in a sophisticated graphical user interface allowing for the efficient inspection and manipulation of the intermediate clustering results. The user has the following main options:

- Merge clusters of genes with apparently too similar prototypes.
- Split clusters in cases wherein a cluster appears to the user to be the union of multiple different time courses.
- Delete clusters in case a cluster is perceived as noise or uninteresting.

We refer to the Computational Methods section in Supplementary Data for formal descriptions and several additional options for users to interfere. Note that all user interference is tracked and can be reverted at any time. Furthermore, the history can be exported as PDF file used for documentation and publication purposes.

### Network enrichment

After clustering, TiCoNE offers several novel network enrichment methods to boost the significance of the derived clusters and put them in a systems biological context. The most straightforward but naive analysis is the extraction of the node-induced subnetworks consisting of the genes presented in a selected cluster of interest. This approach is naive, as it will fail to find connected networks if the genes of a cluster are not directly linked in the network. In a biomedical setting, it is reasonable to assume that not all functionally related objects show a very similar time behavior and end up in the same cluster (e.g., genes in a negative feedback loop). Thus, one may want to allow for a certain number of exception nodes that are not in the selected clusters but connect other objects that are. We use KeyPathwayMiner^17^ to perform this task. It extracts a maximal connected subnetwork consisting only of genes from the selected clusters but a user-given number of exceptions.

### Time course network cross talk

TiCoNE allows for temporal cross talk enrichment by scanning the network for pairs of clusters that are connected more (less) often than expected by chance in a given network. We utilize randomly permuted networks to assess significance levels.

### Phenotype comparison

Different conditions may be compared over time. First, the data for both conditions are treated independently for clustering. After clustering, TiCoNE identifies significantly overlapping clusters of the two conditions and evaluates the similarity of their prototypes. We regard those cluster pairs as most interesting that have a significant overlap and a very similar (or dissimilar) prototype to investigate the commonalities (or the differences) of the conditions. These clusters may afterward be inspected using the aforementioned network enrichment analysis.

### TiCoNE as Cytoscape app and web application

We implemented TiCoNE in a Cytoscape app as well as a feature reduced interactive web application. The TiCoNE Cytoscape app is full featured and includes all approaches described in this article. In this study, TiCoNE is complemented by Cytoscape's network visualization and analysis functionalities. The TiCoNE web application includes only a limited feature set, most importantly the formation of clusters of time series data sets and the visualization and enrichment of identified clusters on a biological network. Temporal cross talk identification and phenotype comparison are not included.

### IAV and RV data

We applied the TiCoNE Cytoscape App to human gene expression data measured with an Affymetrix Human Genome U219 Array containing expression levels for 49,386 probes for 10 time points (baseline and at 2, 4, 8, 12, 24, 36, 48, 60, and 72 h after infection) under three different experimental conditions: BEAS-2B lung cells have been infected with (1) IAV, (2) rhinovirus (RV), or (3) coinfected with both. The data set contained five biological replicates for all time points except the first one, for which it contained six. See Kim et al.^18^ for more details. It is an ideal scenario for TiCoNE's phenotype comparison feature. In this study, we focus on comparing (1) IAV versus (2) RV infection. We mapped the probe set IDs of the data set to Entrez gene IDs. For genes with multiple probe sets measured, we kept only the one with the highest variance over time. The input values have already been log2 transformed such that we only normalized them against the control. Furthermore, we removed genes from the data sets, which were not present in the used interaction network (see the section “Biological network”).

### Clustering of IAV and RV data

We clustered both the expression data of the IAV and the RV infection experiments with CLARA and 20 negative/positive discretization steps into 500 initial clusters. We chose CLARA as it designed to cluster large data sets efficiently. We used the Pearson correlation as similarity function, cluster aggregation function ${f_A} = \mu$, and removed genes not present in the used interaction network (see the section “Biological network”). We then ran automatic clustering iterations until convergence. After this procedure, we performed 1+100=101 iterations for either data set (1 iteration for the initial clustering and 100 iterations for the iterative optimization until convergence). We did not apply any human-augmented operations.

### Biological network

We extracted the human interactome from the Interologous Interaction Database (I2D) protein–protein interaction (PPI) database,^19^ which integrates various other databases of known, experimental, and predicted PPIs. We mapped Uniprot IDs to Entrez gene IDs to be able to integrate the network with our example time series data. After removing duplicate interactions between the same pair of genes, the resulting network contains 199,025 interactions and 15,161 nodes (genes). Note that our TiCoNE approach works with any kind of graph loaded into Cytoscape or to the feature reduced TiCoNE online platform. The use case determines the most appropriate network. In this study, we aimed for finding human response protein complex formation candidates; hence our choice of using the I2D interactome.

## Results

Figure 1 illustrates the structure and unique capabilities of TiCoNE: (1) One may find subnetworks significantly enriched with genes of a similar temporal expression behavior. (2) The same can be applied to several such clusters of genes, that is, multitemporal network expression enrichment. (3) Furthermore, the cross talk between genes over time can be analyzed on a systems level by computing the likelihood of observing more (or less) network interactions between pairs of time patterns by chances, a procedure we call network coenrichment analysis. (4) The TiCoNE software computes empirical *p*-values and cross talk graphs. (5) Finally, one may analyze multiple condition-specific time series experiments to identify overrepresented temporal patterns responding differentially to conditions in multiple experiments.

To demonstrate how TiCoNE can facilitate temporal systems medicine drug target discovery, we analyzed human whole-genome time series transcriptomics data of BEAS-2B lung cells after infection with IAV or RV together with the human protein interactome.^18^ Viral pathogens such as influenza virus pose a severe threat to human welfare.

Antivirals normally attack viral functions, such as antibodies that bind to viral proteins, or drugs that impair viral functions. As an RNA virus, IAV possesses high genetic flexibility, allowing for quick adaptation to selective pressures imposed by these antiviral attacks. The result is that neither a lasting immunization nor an effective therapy has been developed. IAV exploits the host cell after infection by hijacking a variety of fundamental intracellular signaling cascades.^20,21^ Therapeutic target discovery has recently focused on host cell factors needed for or restricting IAV replication, as they are not encoded in the viral genome and the virus cannot easily adapt through mutation to become resistant. Interfering with such factors may thereby allow treating IAV infections independent of the specific virus strain and preventing viral resistance.

The transcriptomics data contain expression levels for 49,386 probes at baseline and over nine time points between 2 and 72 h after infection with five replicates for each time point. TiCoNE clustered the expression data of IAV infection and RV infection experiments into 88 and 71 significant ($p \le 0.05$) clusters, respectively (Supplementary Fig. S1A and B).

For either clustering, we then performed a time course network coenrichment analysis. *p*-Values were derived using a permutation test with 1000 permutations. When comparing the distributions of undirected edge counts between cluster pairs (Supplementary Fig. S2), the clusters of genes after IAV infection represent gene groups that are more strongly interacting (more undirected edges) than the gene clusters after RV infection. We arranged clusters for both conditions according to their most significant cross talk with other clusters ($p \le \ 0.02$) on a timeline (Supplementary Figs. S2 and S3).

In general, IAV cluster pairs with significant cross talk (either depleted or enriched) form a large connected complex along the timeline potentially representing the gene program response cascades to IAV infection over time. In contrast, such significant RV cluster pairs seem to be more fragmented and form multiple separated cluster complexes possibly indicating that no such tightly controlled process of gene program response cascades is activated. Out of the 61 significant gene cluster pairs for the IAV infection, we see more often an enriched number of edges (39/61) than for the 34 significant gene cluster pairs for the RV infection (19/34). These results highlight that both conditions exhibit fundamentally different behavior over time on a systems biology level.

**FIG. 2.**
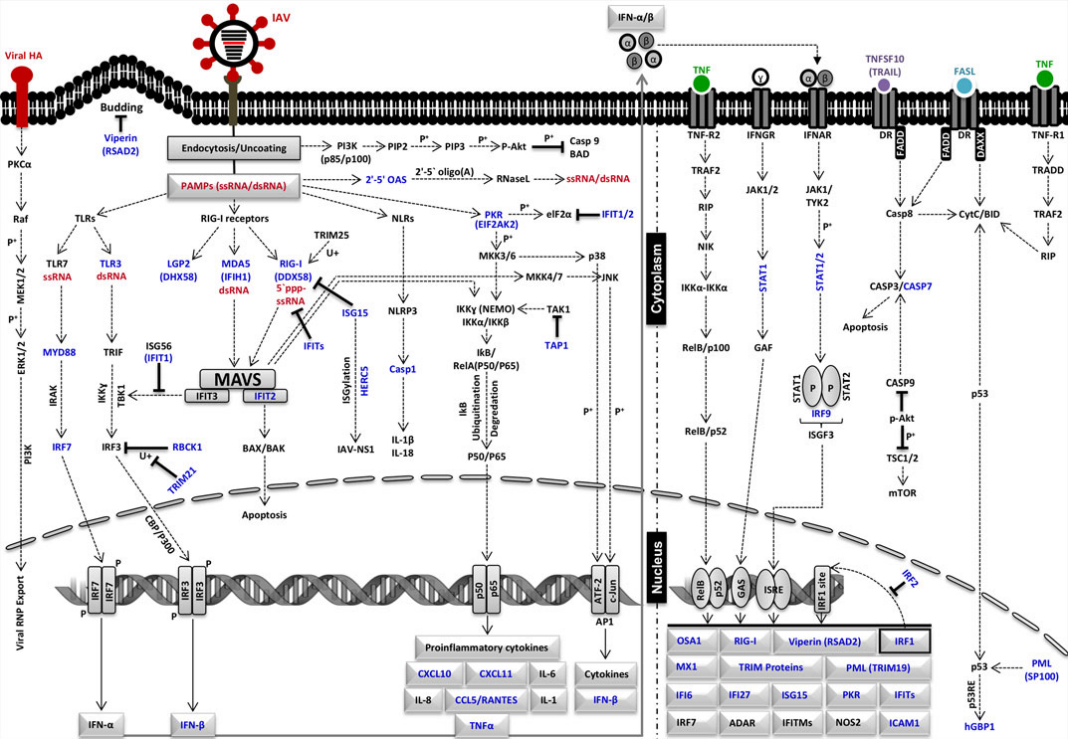
Schematic representation of intracellular signaling pathways that are involved in the regulation of innate immune responses against IAV. IAV infection is associated with unique PAMPs (red), which are recognized by PRRs such as TLRs, RLRs, and NLRs leading to the induction of different signaling pathways such as PI3K-Akt pathway, PKR signaling, OAS1/RNaseL pathway, TLR/RLRs/NLRs signaling, MAPK pathways (JNK pathway/p38 pathway), NF-κB pathways (canonical), and ERK1/2 pathway. After the secretion of interferons (IFN-α, -β) and tumor necrosis factors (TNF-α, -β), the noncanonical NF-κB pathway and STAT1/STAT pathways are stimulated, leading to the expression of several ISGs. At later time points, IAV-induced apoptosis is mainly mediated by TRIAL- and FASL-dependent activation of DRs and subsequent activation of casp3. Factors that were identified by TiCoNE analysis to be significantly enriched in IAV-infected mammalian cells are marked in blue (Supplementary Tables S1 and S2). The defined role of each element is briefly described in Supplementary Table S2. Symbols used in the figure: ⊣, inhibition; ⇢, activation; →, expression; P^+^, phosphorylation; U^+^, ubiquitination. casp3, caspase 3; DRs, death receptors; IAV, influenza A virus; ISGs, interferon-stimulated genes; NLRs, NOD-like receptors; PAMPs, pathogen-associated molecular patterns; PRRs, pathogen recognition receptors; RLRs, RIG-I-like receptor; TLRs, toll-like receptors.

We then compared the systems medicine response between the two infection types (IAV vs. RV) to identify *de novo* pathways in the network that behave differently over time under the two conditions. *p*-Values were derived using a permutation test with 1000 permutations. We identified 30 such pathways (subnetworks) of highly significant size ($p \le \ 0.01$). When enriching for the three most significant pathways ($p \le 0.001$) in the interactome, we find a connected component consisting of 50 genes and 101 interactions (Supplementary Fig. S4). This complex contains 30 genes (60%) that have been annotated as relevant in immune responses to influenza virus infection. Of these 30 genes, 27 have been discussed in the literature, with some also being annotated with a differential behavior between RV and influenza virus infection (Supplementary Table S1). In addition, 15 out of the 50 genes are contained in the Kyoto Encyclopedia of Genes and Genomes (KEGG) pathway for IAV infection (hsa05164).^22,23^ In Figure 2 we have highlighted those gene products, which can currently be attributed to signaling pathways known to be activated upon IAV infection. Their functions within the relevant pathways are summarized in Supplementary Table S2.

Querying the established drug target databases DrugBank and Therapeutic Target Database for the identified genes confirms that TiCoNE recovered several known drug targets (Supplementary Fig. S5), although not (yet) dedicated for IAV infection treatment.

TiCoNE analysis identified several genes, such as phospholipid scramblase 1 (*PLSCR1*), FK506 binding protein like (*FKPBL*), and helicase with zinc finger (*HELZ2*), which were not previously known to be specifically activated upon IAV infection. *PLSCR1* encodes an interferon (IFN)-inducible protein that mediates antiviral activity against DNA and RNA viruses *in vitro* including hepatitis B viruses,^24–26^ vesicular stomatitis virus (VSV),^27^ herpes simplex virus,^28^ and encephalomyocarditis virus.^27^ On the contrary, *PLSCR1* mediates hepatitis C virus entry into host cells.^29^

**FIG. 3.**
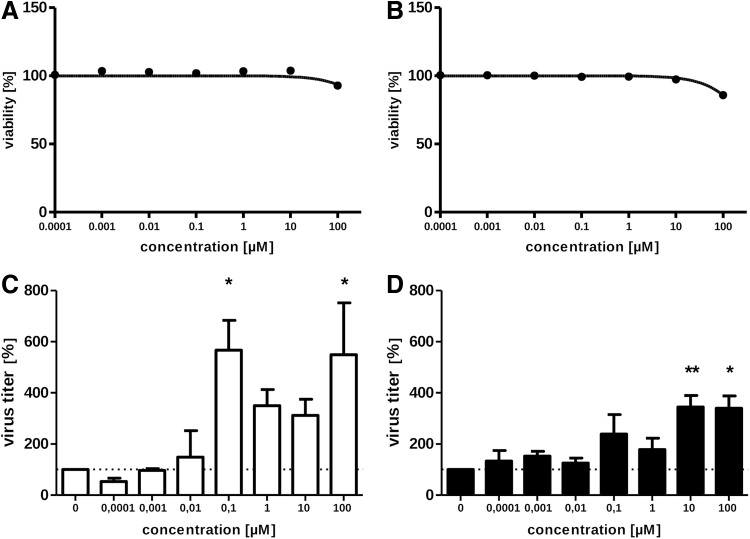
Influence of scramblase inhibitor R5421 on cell viability and IAV replication. A549 **(A)** and BEAS-2B **(B)** cells were treated with R5421 at indicated concentration for 24 h and cell viability was checked using PrestoBlue reagent (MolecularProbes). Data represent means±SEM (*n*=8). A 24-h-old monolayer of A549 **(C)** or BEAS-2B **(D)** cells was infected with PR8 virus at an MOI=1. At 45 min p.i., viral inoculum was removed and cells were overlaid with corresponding medium containing R5421 at indicated concentration or DMSO as a control. Twenty-four hours p.i. supernatants were collected, and virus titers were determined by focus forming assay. Data represent means±SEM (*n*=3). Denoted significance levels are: *p* ≤ 0.05 (*) and *p* ≤ 0.01 (**). p.i., postinfection; SEM, standard error of the mean.

To investigate whether *PLSCR1* activity indeed affects also IAV propagation in an either anti- or proviral manner, we analyzed propagation of the human influenza virus strain A/Puerto Rico/8/1934 (PR8, H1N1) in human lung cells (A549) and in human bronchial epithelial cells (BEAS-2B) in the presence or absence of different concentrations of the *PLSCR1* inhibitor R5421 (Fig. 3). We tested concentrations of R5421 within a range from 0.1 nM to 100 μM for cytotoxicity, and we found them not to be toxic for both cell lines (Fig. 3A, B). Application of R5421 directly after IAV infection concentration dependently increased virus titers for both cell lines (Fig. 3C, D). These data validated that *PLSCR1* is indeed involved in IAV propagation as a negative regulator. *PLSCR1* is implicated in transmitting IFN-induced signals, leading to the expression of IFN-stimulated genes (ISGs),^30^ and it was suggested that the antiviral effect of *PLSCR1* against VSV is correlated with increased expression of specific ISGs. Therefore, phospholipid scramblase 1, which is itself an ISG-encoded protein, is involved in amplifying and enhancing the IFN response through increased expression of a subset of potent antiviral genes.^27^ Also, it was found that primary plasmacytoid dendritic cells (pDCs) from *PLSCR1*-deficient mice produced a lower amount of type-1 IFN than pDCs from the wild-type mice in response to IAV stimulation,^28^ indicating that *PLSCR1* might also be involved in IFN expression and eventually expression of ISGs in IAV-infected cells.

## Discussion and Conclusion

We developed TiCoNE, the first approach to seamlessly identify regions in biomolecular networks that are enriched in genes (or proteins, metabolites) with significantly overrepresented time series behavior or time series coexpression behavior. TiCoNE offers a human-augmented cluster optimization strategy and allows the user to iteratively refine the clustering automatically or visually by applying operations such as adding, deleting, merging, or splitting clusters. Our approach can compare different phenotypes and identify differentially behaving network complexes. TiCoNE computes empirical *p*-values for clusters, phenotype comparisons and network co-enrichment based on different kinds of permutation tests (see Materials and Methods section).

We demonstrate the power of TiCoNE by processing time series gene expression data of human host lung cells, infected with either RV or IAV. In this study, we use our coenrichment analysis approach to construct complexes of clusters along a timeline that are biologically meaningful and may explain the systemic unraveling of host immune response to influenza virus infection. We find specific properties of coenrichments of IAV and RV data clusters, which may help explain the large difference in severity of the two viruses on a systems biology level.

We discovered *de novo* groups of genes that behave consistently but show temporally different behavior under the two conditions. By integrating these candidates with the human PPI network, we discovered a complex of 50 genes, out of which 30 have been previously associated with IAV infection.

Among the identified host genes not previously known to be specifically activated upon IAV infection were *PLSCR1*, *FKPBL*, and helicase with zinc finger (*HELZ2*). Through experimental validation we could confirm that we have identified *PLSCR1* as a novel host factor, which is acting as a negative regulator for IAV infection. These data provide proof-of-principle that a systems medicine approach to analyze the virus and host interactome provides novel and possibly more effective therapeutic approaches in line with network pharmacology^31^ than focusing on the virus only.

With TiCoNE, we provide the first integrated temporal systems medicine drug target identification approach. It extends time series expression data directly to temporal disease-specific subnetworks. It also identifies cross talk between them, and we show that it can identify novel drug targets for IAV infection. TiCoNE is publicly available in the Cytoscape app store^32^ and as feature reduced interactive web application (including screen casts, test data, and online tutorial) at (https://ticone.compbio.sdu.dk).

## Supplementary Material

Supplemental data

## Abbreviations Used

CLARA: Clustering Large Applications
FKPBL: FK506-binding protein like
I2D: Interologous Interaction Database
IAV: influenza A virus
ISGs: interferon-stimulated genes
KEGG: Kyoto Encyclopedia of Genes and Genomes
PAMK: Partitioning Around Medoids
pDCs: plasmacytoid dendritic cells
PLSCR1: phospholipid scramblase 1
PPI: protein–protein interaction
RV: rhinovirus
STEM: Short Time-Series Expression Miner
TiCoNE: time course network enrichment
VSV: vesicular stomatitis virus
