# Supplementary material for: Time-Resolved Systems Medicine Reveals Viral Infection-Modulating Host Targets
Source: Syst Med (New Rochelle). 2019 Mar 28;2(1):1–9. doi: 10.1089/sysm.2018.0013 (PMC6524659; doi:10.1089/sysm.2018.0013)
Supplement: Supplemental data [file Supp_Data.pdf]

Supplementary Data

Supplementary Materials and Methods

Here we briefly outline the details regarding the computational methods and wet laboratory follow-up analysis.

Computational methods

The goal of any partitional clustering method is to partition a given data set $X = \{O_1, \ldots, O_N\}$ with objects O_i into a clustering $\boldsymbol{C}(\boldsymbol{X}) = \{\boldsymbol{c}_1 \ldots \boldsymbol{c}_k\}$, with clusters $\boldsymbol{c} \subseteq \boldsymbol{X}$, where $\bigcup_{\boldsymbol{c} \in \boldsymbol{C}(\boldsymbol{X})} \boldsymbol{c} = \boldsymbol{X}$ and $\bigcup_{\boldsymbol{c_p} \neq \boldsymbol{c_q} \in \boldsymbol{C}(\boldsymbol{X})} (\boldsymbol{c_p} \cap \boldsymbol{c_q}) = \emptyset$. We write $\boldsymbol{C}$ instead of $\boldsymbol{C}(\boldsymbol{X})$, if the data set $\boldsymbol{X}$ is clear from the context. We denote the cluster of an object $\boldsymbol{O} \in \boldsymbol{X}$ in clustering $\boldsymbol{C}$ as $\boldsymbol{C}(\boldsymbol{O})$. Since we focus on genes as objects in this work, we often refer to an object as a gene.

Input and preprocessing. A time series data set consists of time-dependent measurements for a collection of objects (here: genes), where $R \geq 1$ replicates are measured for each object. We formalize the objects $O \in X$ of a time series data set as a collection of R replicates $O = (O^1, \ldots, O^R)$, with each replicate being defined as a time series over T time points: $O^r = \left(O^r_1, \ldots, O^r_T\right), 1 \leq r \leq R, 1 \leq t \leq T$. In the remainder, the term "object" includes all of its replicate time series.

The preprocessing of raw data is a highly setting-specific task, and it should thus be performed by the user before clustering in time course network enrichment (TiCoNE). We provide two basic data preprocessing operations:

- Remove baseline objects, that is, $X^* = X \setminus \{O \in X | \exists\, r \in 1 \ldots R : \overline{\sigma(O^r)} < \sigma^*\}$, where σ^* is a user-given threshold for the average standard variance of objects to keep.
- Remove objects O with low agreement between replicates: $X^* = X \setminus \{O \in X | A(O) < A^*\}$, where A^* is a user-given threshold for the replicate agreement of objects to keep, and $A(O)$ the replicate agreement of object O as defined in the next section.

Similarities. The objective of clustering is to group the genes in such a way that the time courses in one cluster are more similar to each other than to the time courses of other clusters. We denote the similarity of two genes as $S\left(O_i, O_j\right) = \frac{1}{R^2} \sum_{r=1}^{R} \sum_{r=1}^{R} S\left(O^r_i,\ O^r_j\right)$. We support two measures to assess the similarity $S\left(O_i, O_j\right)$ between time courses of two genes $O_i, O_j \in X$: (1) the (negative) Euclidean distance $S_E\left(O_i, O_j\right)$ and (2) the (scaled) Pearson correlation $S_P\left(O_i, O_j\right)$. We define the negative Euclidian distance of two vectors $x, y \in \mathbb{R}^T$ as $S_E(x, y) = -d_E(x, y) = -\sqrt{\sum_{t=1}^{T} (x_t - y_t)^2}$ and the scaled Pearson correlation as $S_P(x, y) = \frac{\rho(x, y) + 1}{2}$.

The former assesses the similarity of the time courses according to the magnitudes, the latter according to their shape. We denote the case $S(O, O) = A(O)$ and call it the agreement of object O's replicates. The similarity of an object O to a prototype P can be defined as $S(O, P) = \frac{1}{R} \sum_{r=1}^{R} S(O^r,\ P)$.

Prototype discretization. To give the user control over the tradeoff between sensitivity, that is, detecting more and smaller clusters, and selectivity, that is, finding less clusters of random noise, cluster prototypes are discretized and can only assume values $x \in N^- \cup N^+ \cup \{0\}$, where N^- is a set of $n^- = |N^-|$ negative discretized values, and N^+ is a set of $n^+ = |N^+|$ positive discretized values. We define the sets of discretized negative and positive values as follows:

$$N^- = \left\{min, \frac{(n^- - 1)}{n^-} \cdot min, \frac{(n^- - 2)}{n^-} \cdot min, \ldots, \frac{1}{n^-} \cdot min\right\}$$

$$N^+ = \left\{\frac{1}{n^+} \cdot max, \frac{2}{n^+} \cdot max, \ldots, max\right\},$$

where min and max denote the minimum and maximum input values observed for any gene at any time point and replicate.

Iterations, initial and final clustering. TiCoNE performs iterations $i \in \{1 \ldots F\}$, where F denotes the final iteration. We denote the clustering in iteration i as $C^i = \left\{c^i_1, \ldots, c^i_{|C^i|}\right\}, 1 \leq i \leq F$, where we refer to C^F as the final clustering. If a clustering C is denoted without iteration, we assume it to correspond to the

last performed iteration i' in the context, that is, it is implied that $C = C^{i'}$. TiCoNE uses one of the following integrated clustering methods to find an initial clustering C^1 with a user-specified number of clusters $|C^1|$: Clustering Large Applications (CLARA),[S1] k-means,[S2] Partitioning Around Medoids (PAMK),[S3] Short Time-series Expression Miner (STEM),[S4] or transitivity clustering.[S5] For all clusters $c \in C^1$ new prototypes $\langle c \rangle$ are calculated. C^1 can then be inspected and iteratively refined in later iterations, resulting in clusterings $C^2, C^3, \ldots, C^F$.

Prototypes and clustering optimization iterations. TiCoNE follows a prototype-based approach[S6,S7] similar to k-means.[S2] With such approaches each cluster $c \in C$ is represented by a prototype $\langle c \rangle$, which is a representation of objects (i.e., genes) $O \in c$. Usually, $\langle c \rangle$ is derived from all $O \in c$ using some aggregation function f_A. We define $\langle c \rangle = (f_A(c,1), \ldots, f_A(c,T))$ with T being the number of time points. We provide three aggregation functions in TiCoNE, where $r \in \{1 \ldots R\}$ and $t \in \{1 \ldots T\}$:

- The mean: $f_A(c,t) = \overline{\{O_t^r | O \in c\}}$
- The median: $f_A(c,t) = median\{O_t^r | O \in c\}$
- The most central object: $f_A(c,t) = \overline{\left\{ O_t^{*^r} | O^* = \underset{O \in c}{\operatorname{argmax}} \sum_{O_k \neq O \in c} S(O, O_k) \right\}}$.

At any stage during the clustering process, the user can let TiCoNE optimize the current clustering by either performing (1) one optimization iteration or (2) optimization iterations until convergence. Assume, we are in iteration i_s with clustering C^{i_s}. Execution of one optimization iteration then consists of performing the following two steps, where $O \in X$:

1. Reassign genes to the most similar prototype, that is, $C^{i_s+1}(O) = \operatorname{argmax}_{c \in C^{i_s}} \mathrm{S}(\mathrm{O}, \langle c \rangle)$, where we break ties randomly.
2. Recalculate prototypes $\langle c^{i_s+1} \rangle$ based on the new genes.
   a. Remove empty clusters, that is, $C^{i_s+1} = C^{i_s} \setminus \{c \in C^{i_s} | c = \emptyset\} = \emptyset$ unless c is marked as "keep."
   b. Clusters with identical prototypes are merged.

Iterations until convergence stops as soon as the following condition holds: $C^{i'+1} = C^{i'} \vee i' \geq i_s + 100$, that is, either the clustering was not changed in the iteration, or we performed 100 iterations.

Human augmentation operations. With TiCoNE we offer a human augmented clustering approach, which implies that the user may (but does not have to) manually influence the clustering outcome. In contrast to the fully automated clustering approaches, TiCoNE makes better use of field-specific knowledge that a fully automated procedure is not aware of. Human-augmented algorithms have been presented in several disciplines, such as robotics,[S8] linguistics,[S9] and also bioinformatics.[S10] In TiCoNE, the user can modify a clustering in any of the following ways, where i' denotes the current iteration:

- Merge clusters: A set of selected clusters $\hat{C} \subseteq C$ can be merged together into a new cluster c^*. The new cluster is defined as $c^* = \cup \hat{C}$ and the new clustering as $C^{i'+1} = (C^i \setminus \hat{C}) \cup \{c^*\}$. The cluster's prototype $\langle c^* \rangle$ is initialized appropriately. This allows to reduce duplicated clusters, when the number of clusters was set too high.
- Split clusters: A cluster $c \in C^{i'}$ can be split into a set of clusters $\hat{C}$ where $C^{i'+1} = (C^{i'} \setminus \{c\}) \cup \hat{C}$. This can be done in two different ways: (1) use objects $O_1 \neq O_2 \in c$ with $\operatorname{argmin}_{O_1, O_2} S(O_1, O_2)$ to calculate prototypes $\langle \hat{c_1} \rangle = \overline{O_1^r}$ and $\langle \hat{c_2} \rangle = \overline{O_2^r}$ for two new clusters $\hat{C} = \{\hat{c_1}, \hat{c_2}\}$ or (2) objects $O \in c$ can be clustered into a user-specified number of smaller clusters using one of the available clustering methods. This allows to improve overall clustering prototype–object similarities, when the number of clusters was set too low.
- Keep a prototype: A cluster c can be marked as "keep", here denoted as $\vec{c}$. If a cluster has been marked as keep in iteration i' it holds that $\overrightarrow{c^{i'}} = \overrightarrow{c^{i'+1}}$ and $\overrightarrow{c^{i'}} \in C^{i'} \Leftrightarrow \overrightarrow{c^{i'+1}} \in C^{i'+1}$, even if $\overrightarrow{c^{i'}} = \emptyset$. The "keep" flag can always be revoked in later iterations.
- Add cluster with predefined prototype: A cluster c with a user-specified prototype $\langle c \rangle$ can be added to the clustering. Initially it holds that $c = \emptyset$. To prevent immediate removal of c from C, we set it to keep, that is, $c = \vec{c}$.
- Delete cluster or its objects: The user can delete a cluster $c \in C$ and (1) delete its genes from the data set and its prototype from the clustering, (2) delete its prototype but keep its genes, (3) delete its genes but keep its prototype, or (4) delete its least-fitting genes, that is, $c^{i'+1} = c^{i'} \setminus \{O \in c^{i'} | S(O, \langle c^{i'} \rangle) < S^*\}$, where S^* is a user-defined similarity threshold.

- Handle least-fitting objects: The user has the possibility to remove genes from the clustering that are least similar to their respective prototypes, that is, it holds that $\forall c^{i'} \in C^{i'} : c^{i'+1} = c^{i'} \setminus \{O \in c^{i'} | S(O, \langle c^{i'} \rangle) < S^*\}$, where S^* is a user-defined similarity threshold. The deleted genes can either be (1) completely removed from the clustering or (2) clustered into a user-specified number of new clusters.

Clustering history and reproducibility. Throughout the clustering process, TiCoNE keeps a history of all performed steps. This includes automatically performed iterations, as well as human augmentations. At any time, the user can inspect clusterings $C_1, \ldots, C_F$ and, if desirable, revert the current clustering to a previous stage. The history can be exported for publication documenting the clustering process.

Statistics

TiCoNE makes use of different fitness scores in different contexts to calculate empirical p-values, for example for clusters. If multiple fitness scores should be used to derive an overall p-value, a p-value is calculated for each fitness score independently, which are then combined into an overall p-value as described in the following.

For an observed instance (e.g., cluster), the p-value for one specific fitness score is calculated by comparing the original fitness against fitness scores of instances from a background distribution. The p-value is defined as the number of times we observe fitness scores at least as extreme as the original score.

Let FS be a fitness score, let $Z^* = (z_1^*, \ldots, z_Q^*)$ be the permuted instances, and let $1(b)$ be the indicator function, defined as follows:

$$\mathbf{1}(b) = \begin{cases} 1, & \textit{if } b \\ 0, & \textit{otherwise} \end{cases} \quad , \ b \in \{\text{true}, \text{false}\}.$$

In a one-sided setting, the p-value for FS and z is then defined as

$$p(z; FS) = \frac{1}{Q} \cdot \sum_{z^* \in Z^*} \mathbf{1}(FS(z^*) \geq FS(z)).$$

And in a two-sided setting as

$$p(z; FS) = \frac{1}{Q} \cdot \left(2 * min \left(\sum_{z^* \in Z^*} \mathbf{1}(FS(z^*) > FS(z)), \sum_{z^* \in Z^*} \mathbf{1}(FS(z^*) < FS(z)) \right) + \sum_{z^* \in Z^*} \mathbf{1}(FS(z^*) = FS(z)) \right),$$

we combine p-values of multiple fitness scores $FS_1, \ldots, FS_R$ for an instance z by using a function CO: $p(z) = CO(p(z; FS_1), \ldots, p(z; FS_R))$. TiCoNE combines p-values with $CO = \prod max(p(z; FS_i), \ PSEUDO)$, where PSEUDO refers to a pseudo count constant (default: 0.0001).

Generating background distributions. TiCoNE provides multiple ways of generating background distributions for p-value calculations.

1. Permuting clusterings (PC): We provide several ways of creating random clusterings: (PC1) Take a given clustering, shuffle the values of the cluster prototypes over time, and reassign objects to shuffled prototypes. (PC2) Generate k random prototypes and assign objects to those. (PC3) Take a given clustering and randomly assign objects to the cluster prototypes while maintaining the cluster sizes. (PC4) Take a given clustering and randomly assign objects to the cluster prototypes while trying to maintain the sum of the node degrees in each cluster, given the network. We sort all objects according to the node degrees in the network (descending). We then greedily assign the objects from top to bottom to random cluster, but only those that have not reached the sum of degrees of the original cluster. Note that this method yields p-values comparable with methods permuting the network using edge crossover but with substantially reduced computing time.
2. Permuting time series data (PD): We employ two approaches to create a permuted version X^* of a given time series data set X: (PD1) Object-wise: We shuffle the values of an object across time points, that is, we reassign each value once to a random time point without replacement. Each gene is permuted independently, and thus it keeps the same distribution of values. If a gene has several replicates, they are shuffled in the same order. (PD2) Globally: Values are shuffled across all objects at the same time without replacement. Here the value distributions of the gene can change. Several replicates of the same gene are shuffled in the same order. In both approaches, each gene and time point is shuffled exactly once.
3. Permuting networks: We create a permuted version $G^* = (V^*, E^*)$ of a given network $G = (V, E)$

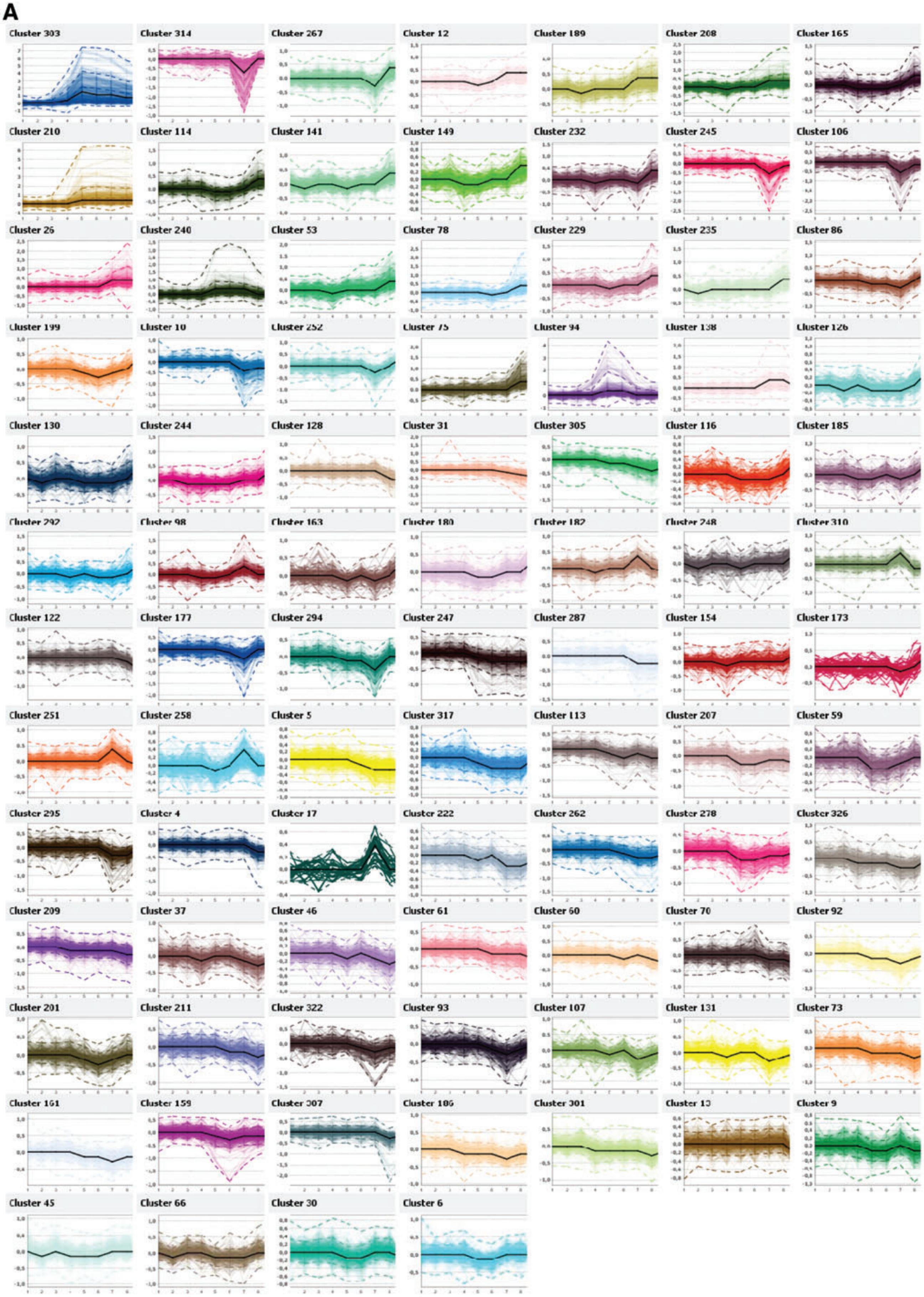

SUPPLEMENTARY FIG. S1. Temporal gene expression patterns after IAV and RV infection. Significant clusters ($p \leq 0.05$) of the final clustering of the **(A)** IAV and **(B)** RV data set. Clusters are sorted by prototype standard variance. Note the individual y-axis scaling. IAV, influenza A virus; RV, rhinovirus.

(*Figure 1 continued*)

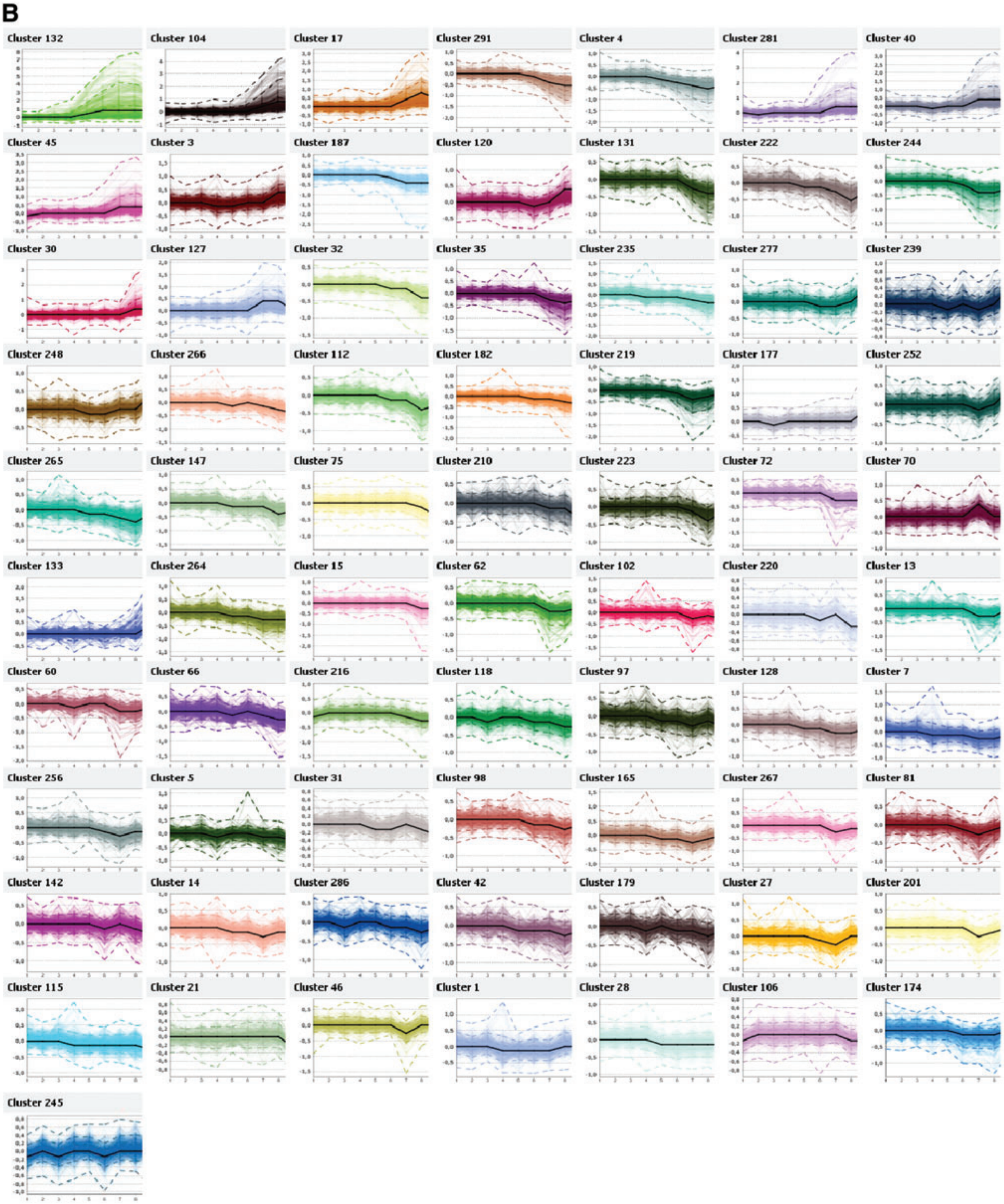

SUPPLEMENTARY FIG. S1. (Continued).

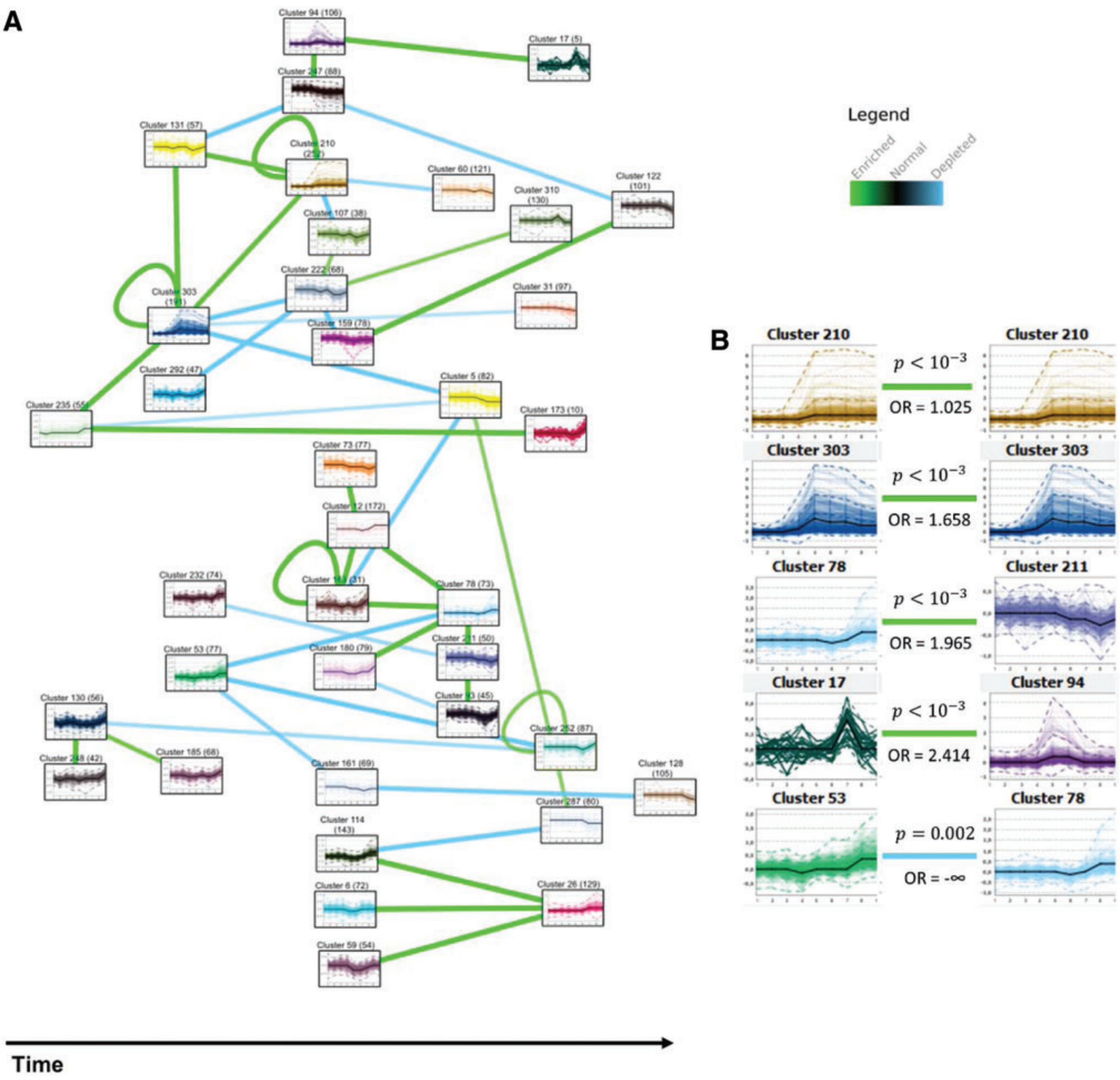

SUPPLEMENTARY FIG. S2. IAV infection temporal cross-talk. **(A)** Temporal cross-talk representation of temporal expression patterns after IAV infection expressed by genes that interact more (less) often than expected by chance. The edge colors correspond to log odds scores. Only edges with $p \leq 0.02$ are shown. Nodes are labeled with the corresponding cluster number and their size (number of objects) in brackets. Clusters are arranged along a timeline regarding their peak variance. **(B)** Top 5 cluster pairs with the most significant cross talk. Pairs of clusters are depicted together with the log OR and the empirical *p*-value (*p*) of their cross talk. *p*-Values were derived using a permutation test with 1000 permutations. OR, odds ratio.

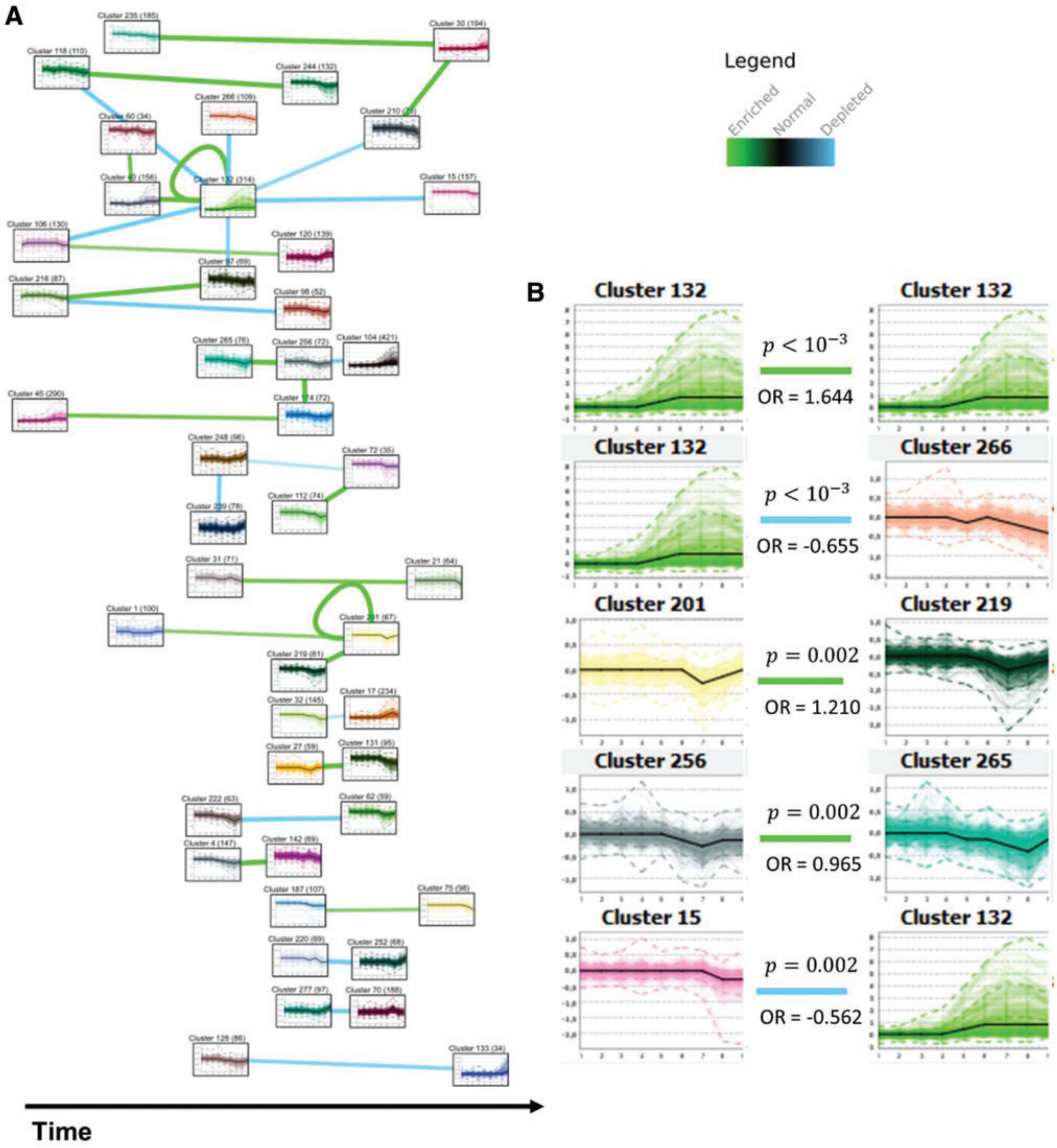

SUPPLEMENTARY FIG. S3. RV temporal cross-talk. **(A, B)** Temporal cross-talk representation of temporal expression patterns after RV infection; analogous to Supplementary Figure S2.

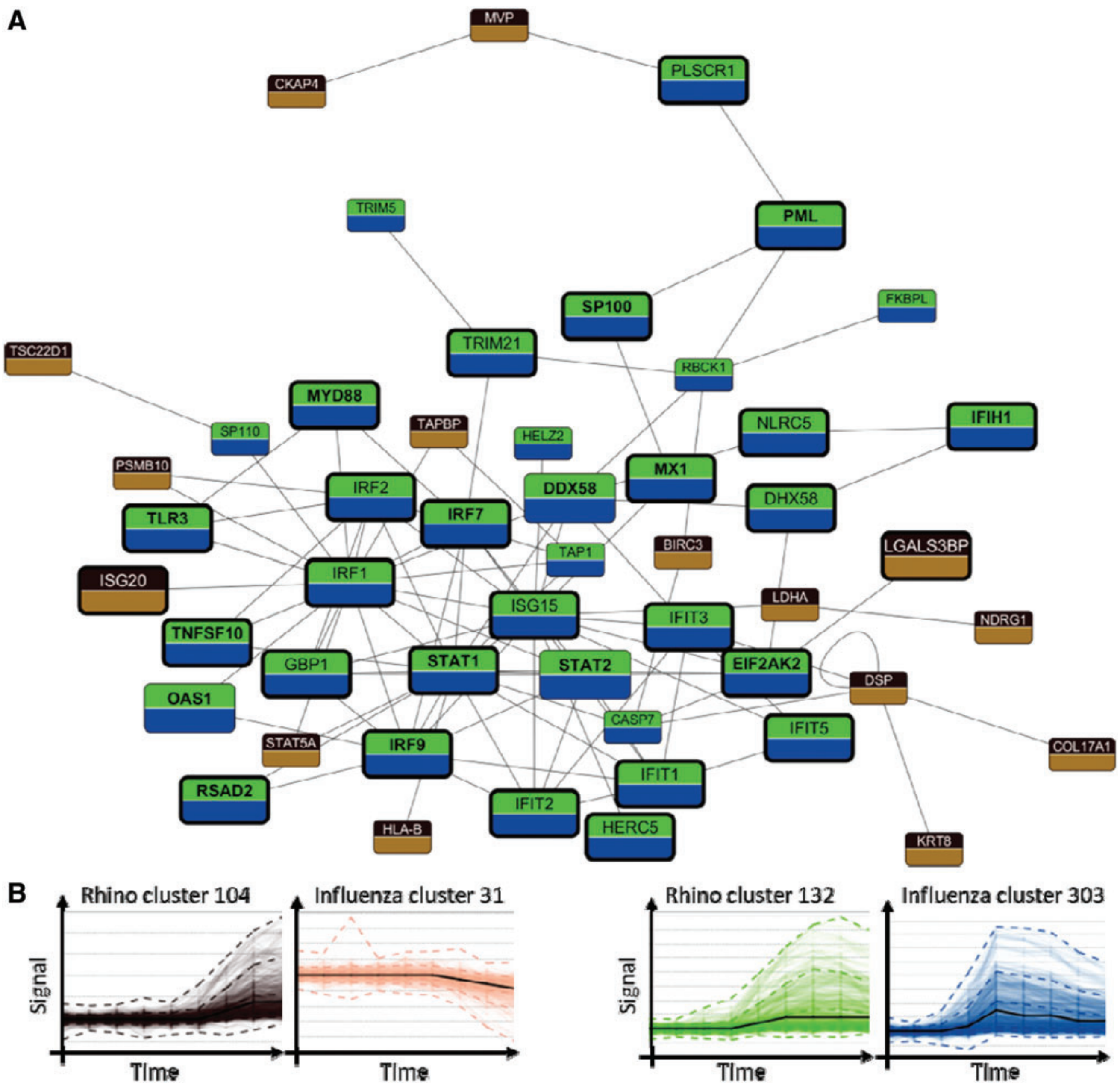

SUPPLEMENTARY FIG. S4. Phenotype comparison between RV and influenza virus response patterns. **(A)** Largest connected subnetwork enriched with genes of the two most significant cluster pairs (size: 50 genes). Node colors correspond to the genes' respective cluster in the RV (upper half) and IAV data sets (lower half). Larger nodes correspond to 30 known IAV-related genes, where we consider two kinds of annotations: bordered nodes correspond to genes linked to IAV infection in the literature; genes with bold font are contained in the IAV KEGG pathway. **(B)** The two most different pairs of temporal gene cluster pairs of RV versus IAV infection response. KEGG, Kyoto Encyclopedia of Genes and Genomes.

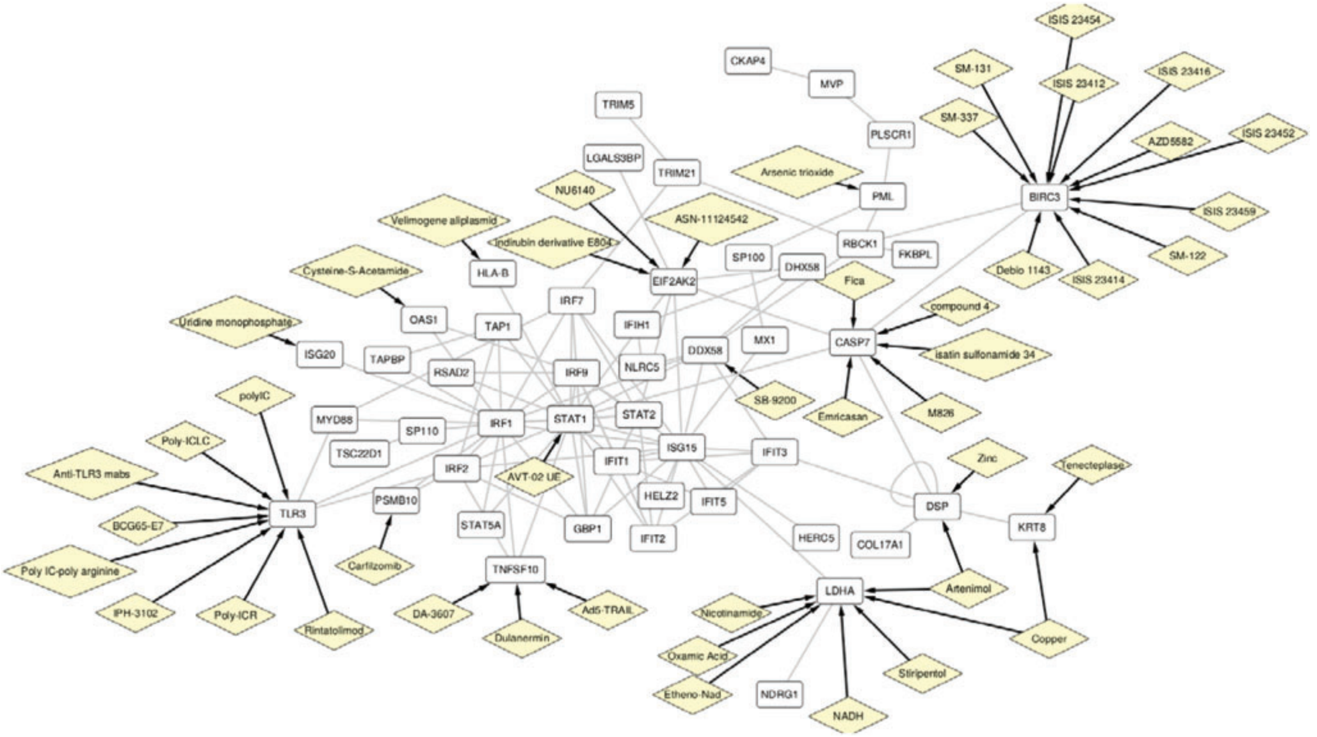

SUPPLEMENTARY FIG. S5. Genes with differential expression between IAV and RV infection identified by TiCoNE are known drug targets. Some of the genes identified by TiCoNE because of their differential expression between the two conditions are known drug targets in DrugBank and TTD. White boxes correspond to targets, orange diamonds correspond to drugs. TiCoNE, time course network enrichment; TTD, Therapeutic Target Database.

by performing edge crossovers (also called edge swaps). Let an edge be defined as $e = (v_1, v_2) \in E$, where $v_1, v_2 \in V$. We call v_1 the source of e and v_2 the target of e. Given two randomly chosen edges $e_1 = (v_1, v_2)$, $e_2 = (v_3, v_4) \in E$, we swap their targets and thus $E = E \setminus \{e_1, e_2\} \cup \{(v_1, v_4), (v_3, v_2)\}$. In case of undirected networks, source and target of an edge need to be treated interchangeably when swapping. To ensure that the permuted network is sufficiently randomized, we swap $\frac{|E|}{2} \cdot c$ times, where $c = \ln(10^7)$.[S11]

Significance of clusters. TiCoNE calculates empirical p-values for clusters based on the permutation scheme already outlined. The fitness scores for cluster p-values are the number of objects, $FS_1 = |c|$, and the average object–prototype similarity, $FS_2 = \overline{s(c)} = \frac{1}{|c|} \sum_{O \in c} S(O, \langle c \rangle)$. Thus, cluster p-values approximate the probability of observing a cluster c^* at random with $|c^*| \geq |c|$ and $\overline{s(c^*)} \geq \overline{s(c)}$. In our example study, we use permutation method PC2 to generate random clusterings.

Network enrichment of clusters. When a data set has been clustered, TiCoNE allows to identify connected regions in a network that are enriched in one or multiple clusters. We provide two ways of identifying subnetworks $\hat{G} = (\hat{V}, \hat{E}) \subseteq G = (V, E)$ enriched in genes of a selected set of clusters $\hat{C} \subseteq C$: (1) Extracting node-induced subnetworks with $\hat{V} = \cup \hat{C}$ and $\hat{E} = \{\hat{V} \times \hat{V} \in E\}$. Such networks are algorithmically easy to compute. However, this approach will fail to find connected networks if the genes of a cluster are not directly linked in the network. (2) In a biomedical setting it is reasonable to assume that not all functionally related objects show a very similar time behavior and end up in the same cluster. Thus, we may want to allow for a certain number of exception nodes that are not in the selected clusters but connect other objects that are. We use KeyPathwayMiner[S12] to perform this

Supplementary Table S1. Genes Contained in the Complex Discovered by Time Course Network Enrichment with Differential Behavior Upon Influenza A Virus and Rhinovirus Infection

Gene	Annotated to IAV	Annotated differentially to IAV vs. RV	KEGG pathway
BIRC3			
CASP7			
CKAP4			
COL17A1			
DDX58			x
DHX58 (LGP2)	x[S14,S15]		
DSP			
EIF2AK2	x[S16,S17]		x
FKBPL			
GBP1	x[S18,S19]		
HELZ2			
HERC5	x[S20,S21]		
HLA-B			
IFIH1	x[S22,S23]		x
IFIT1	x[S24]		
IFIT2	x[S25]		
IFIT3	x[S25]		
IFIT5	x[S25]		
IRF1	x[S26]		
IRF2	x[S27]		
IRF7	x[S28]		x
IRF9	x[S29]	x[S29]	x
ISG15	x[S30]		
ISG20	x[S31,S32]		
KRT8			
LDHA			
LGALS3BP	x[S33]		
MVP			
MX1	x[S34,S35]		x
MYD88	x[S36]		x
NDRG1			
NLRC5	x[S37]		
OAS1			x
PLSCR1	x[S38]		
PML	x[S39,S40]		x
PSMB10			
RBCK1			
RSAD2	x[S26,S40]		x
SP100	x[S41]		x
SP110			
STAT1	x[S42]	x[S43–S45]	x
STAT2			x
STAT5A			
TAP1			
TAPBP			
TLR3	x[S46]		x
TNFSF10	x[S47]		x
TRIM21	x[S48]		
TRIM5			
TSC22D1			

The 50 genes discovered by TiCoNE's phenotype comparison analysis that show differential behavior upon IAV and RV infection. Genes are marked in the respective columns, if they have been annotated in the literature as IAV related, if they have been annotated in the literature with a differential behavior between IAV and RV infection, or if they are contained in the IAV KEGG pathway.

IAV, influenza A virus; KEGG, Kyoto Encyclopedia of Genes and Genomes; RV, rhinovirus; TiCoNE, time course network enrichment.

task. It extracts a maximal connected subnetwork consisting only of genes from the selected clusters but a user-given number of exceptions.

Phenotype comparison. If biological entities have been analyzed under more than one experimental condition and are given as two data sets X and Y, the first step is to cluster the objects for each setting separately and then compare the resulting clusterings $C(X)$ and $C(Y)$ for differentially clustered object (e.g., gene) sets. TiCoNE can identify such objects by comparing each cluster $c_1 \in C(X)$ with each cluster $c_2 \in C(Y)$. It then presents these clusters with their normalized number of common objects $NCO(c_1, c_2) = \frac{CO(c_1, c_j)}{\min(|c_1|, |c_j|)}$ and the similarity of their prototypes $S(\langle c_1 \rangle, \langle c_2 \rangle)$.

We assume that a cluster pair (c_1, c_2) from two different phenotypes is interesting, if c_1 and c_2 have more objects in common than expected by chance for a pair of random clusters with a prototype similarity at most this high. We calculate p-values for each pair of clusters $(c_1, c_2) \in C(X) \times C(Y)$ using our permutation scheme and the fitness score $FS = NCO(c_1, c_2) \times \mathrm{S}(\langle c_1 \rangle, \langle c_2 \rangle)$. For our example study, we generate random clusterings using permutation method PC2.

Network enrichment of differential phenotype. Once phenotypes have been compared, TiCoNE can identify subnetworks that are enriched in genes with a differential time behavior. Again, refer to the Supplementary Materials and Methods section for detailed descriptions of available approaches.

Wet laboratory validation

Cell lines. The human lung adenocarcinoma cell line A549 and the human bronchial epithelial cells BEAS-2B were purchased from ATCC (Manasses, VA). Madin–Darby canine kidney II (MDCK-II) cells were obtained from the cell culture collection of the Institute of Medical Virology, University Giessen, Germany. A549 and MDCK-II cells were maintained in Dulbecco's modified Eagle medium (DMEM; Life Technologies), containing 10% heat-inactivated fetal calf serum (FCS; Life Technologies) and antibiotics at 37°C, 5% CO_2. BEAS-2B cells were cultivated in bronchial epithelial cell growth medium (BEGM; Lonza, Switzerland), supplemented with 10% FCS and antibiotics at 37°C, 5% CO_2.

Supplementary Table S2. Influenza A Virus Infection Genes' Functions

Gene symbol	Protein name	Role in IAV infection	Reference
CASP1	Caspase 1	Virus-induced inflammasome complex, which induces auto-activation of caspase-1 to proteolytically activate pro-IL-1β and pro-IL-18 and thereby inaugurates the inflammatory form of cell death, namely pyroptosis.	S49,S50
Casp7	Caspase 7	IAV activation of caspase 7 induces apoptosis<br>IAV-induced caspase enlarges the nuclear pores, thereby facilitating nucleo/cytoplasmic translocation processes including nuclear transport of viral ribonucleprotein complex (vRNP)	S51,S52
CXCL10/11	C-X-C motif chemokine 10/11	Monocyte-attracting chemokines, which have unique activity in host defense	S53
DDX58 (RIG-I)	DExD/H-box helicase 58 (retinoic acid-inducible gene 1 protein)	Recognizes viral dsRNA and thereby induces a variety of innate immune responses through MAVS-dependent signaling	S54, S55
HERC5	Probable E3 ubiquitin–protein ligase HERC5	Attenuates IAV by catalyzing ISGylation of the viral NS1 protein, which impedes with the functionally important homodimerization of NS1.	S56
hGBP1	Guanylate binding protein 1	IFN-inducible protein plays an important antiviral role	S18
ICAM1 (CD54)	Intercellular adhesion molecule 1	IFN-inducible antiviral protein, which represses IAV replication in airway epithelial cells	S57
IFI6/IFI27	Interferon alpha inducible protein 6/interferon alpha inducible protein 27	IFN-inducible proteins upon IAV infection, which are clinically useful as biomarkers to discriminate between viral and bacterial infections	S58,S59
IFIT1–3, IFIT5	Interferon-induced protein with tetratricopeptide repeats 1–3, 5	IFIT1 and IFIT2 directly bind eIF3 to control the transcription of the invading viral genes.<br>The cytoplasmic IFIT1/IFIT2/IFIT3 complex or IFIT5 alone sense and breakdown the 5′ triphosphate double-stranded RNA (5′ ppp-dsRNA).<br>The IFIT3 bridges TBK1 to MAVS in mitochondria, to subsequently synergize the activation of IRF3 and NF-κB.<br>The IFIT2 interacts with MITA and induces apoptosis through BAX/BAK activation.<br>The IFIT1 negatively regulates the cellular antiviral response by disrupting the interaction between TBK1, MAVS, and MITA	S48,S54,S60–S62
IFNB1	Interferon beta 1	Type-I IFN plays a pivotal role to limit IV replication<br>Secreted IFNB1 induces a variety of ISGs to augment the innate immune response against the invading pathogen	S63
IRF1	Interferon regulatory factor 1	IRF-1 activates the transcription of different ISGs, such as RIG-I and viperin	S64
IRF2	Interferon regulatory factor 2	Feedback inhibitor of IRF-1-mediated effects	S64,S65
IRF7	Interferon regulatory factor 7	Triggers type 1 IFN expression	S63,S66
IRF9	Interferon regulatory factor 9	Induces ISGs through ISGF3 formation	S67
ISG15	Interferon-stimulated gene 15	An early proviral role by downregulating the cellular innate immune response.<br>Antiviral role in IAV-infected cells through ISG15-mediated modification of the K41 residue, which is important for RNA binding efficiency and nuclear localization of NS1 protein of IAV.	S56,S68
LGP2 (DHX58)	Laboratory of genetics and physiology-2 protein	Negative regulator of antiviral signal transduction processes by sequestering dsRNA from RIG-I<br>Decreasing IRF3 activation<br>Downregulation of the IAV -triggered detrimental inflammatory response	S15,S69
MDA5 (IFIH1)	Melanoma differentiation antigen 5	Activates MAVS-dependent signaling restricting viral infection and pathogenesis	S55
MX1	Myxovirus resistance protein 1	IFN-inducible protein that inhibits IAV by interfering with the transcriptional function of the vRNP complex assembly by binding to viral nucleoprotein	S70
MYD88	Myeloid differentiation primary response 88	Regulates the activation of IRF7 and thereby type-1 IFN induction	S36,S71
NLRC5	NOD-like receptor family CARD domain containing 5	NLRC5 is a negatively regulating interferon activity through direct interaction with RIG-I, MDA5, and IKKα/IKKβ (NF-κB pathway) to block their activation and associated downstream cascades	S72
OAS1	OAS1 2′-5′-oligoadenylate synthetase 1	OAS1 activates latent RNase L, which degrades viral and cellular ssRNAs, and thereby inhibits protein synthesis and viral growth. Induces apoptosis.	S73–S76

(*continued*)

Table ST2. **(Continued)**

Gene symbol	Protein name	Role in IAV infection	Reference
PKR (EIF2AK2)	dsRNA-activated protein kinase	Phosphorylates the α-subunit of EIF2α, resulting in inhibition of total cellular and viral protein synthesis.<br>In addition, PKR has a role in the induction of the cytokines TNF-α, IL-8, and RANTES through activation of the p38 MAPK signaling cascade.<br>NF-κB is present as a complex with its inhibitor IκB. PKR regulates the NF-κB pathway through activation of the IκB inhibitor, which results in its degradation and liberation of NF-κB.	S26,S77–S80.
PML or TRIM19	Promyelocytic leukemia	Negative influence on IAV replication *in vivo*<br>Key component of NBs<br>PML-NBs regulate the recruitment and activation of p53, enhancing apoptosis as a response to stress conditions	S41,S81
RBCK1	E3 ubiquitin ligase RBCC protein interacting with PKC1″	IAV infection induces the RBCK1, which catalyzes the RBCK1-mediated ubiquitination and degradation of IRF3	S82
SP100	SP100 nuclear antigen	NB-associated protein	S83
STAT1/STAT2	Signal transducer and activator of transcription 1/signal transducer and activator of transcription 2	Active/phosphorylated STAT1/STAT2 engages with IRF9 to form the heterotrimeric transcriptional factor complex ISGF3 to induce ISGs expression<br>The active/phosphorylated STAT1 molecules homodimerize forming the GAF, which localizes to the nucleus to induce different ISGs through its association with the IFN-γ responsive GAS.	S67,S84,S85
TAP1	Transporter associated with antigen processing 1	TAP1 inhibits NF-κB signaling by suppressing the TAK1 phosphorylation and consequently promotes IAV replication	S86
TLR3	Toll-like receptor 3 (cytosolic sensor)	Antiviral binding to dsRNA to enhance downstream IRF3 induction and type 1 IFN expression	S46,S87
TRAIL (TNFSF10)	TNF-related apoptosis-inducing ligand	A member of the TNF family of ligands, which initiates apoptosis upon binding to the DRs<br>Plays an important role in the immune response to virus infection	S47,S88
TRIM21	Tripartite motif-containing protein 21	Antiviral activity by sustaining the IRF3 activation	S89,S90
TRIM5	Tripartite motif containing 5	TRIM5α restricts HIV in a similar way by which TRIM22 exerts its antiviral activity against IAV	S91
Viperin (RSAD2)	Virus inhibitory protein (radical SAM domain-containing 2)	IFN-inducible protein that disrupts the lipid rafts in infected cell plasma membrane to block viral budding	S92

Gene products (and their functions) that can currently be attributed to signaling pathways known to be activated upon IAV infection (see also Figure 3 in main document).

DRs, death receptors; dsRNA, double-stranded RNA; EIF2α, eukaryotic translation initiation factor 2α; GAF, gamma activation factor; GAS, gamma activation sequence; IFN, interferon; ISGs, interferon-stimulated genes; ISGF3, IFN-stimulated gene factor 3; MAVS; NBs, nuclear bodies; ssRNAs, single-stranded RNAs; TNF, tumor necrosis factor.

Virus infection and titration. MDCK-II cells were infected with influenza virus A/Puerto Rico/8/34 (PR8, H1N1) at a multiplicity of infection (MOI) of 0.01 in phosphate-buffered saline containing 0.2% bovine albumin (PBS/BA [PAA, Germany], 1 mM $MgCl_2$, 0.9 mM $CaCl_2$, 100 U/mL penicillin, and 0.1 mg/mL streptomycin [P/S]). Inoculum was replaced by DMEM/BA (DMEM containing 0.2% BA, P/S, 1 μg/mL of L-1-tosylamide-2-phenylethyl chloromethyl ketone [TPCK]-treated trypsin; Sigma Aldrich). Virus titer was determined at the indicated time points by focus forming assay as previously described.[S13] In brief, MDCK-II cells grown in 96-well plates overnight at 37°C, 5% CO_2 to ~90% confluence were washed once with PBS++ (PBS containing 1 mM $MgCl_2$, 0.9 mM $CaCl_2$) and subsequently inoculated (in duplicates) with 50 μL (per well) of fivefold serial dilutions (total of eight times) in PBS/BA of the respective virus samples. Inoculum was aspirated and 150 μL Avicel medium (DMEM/BA, containing, 1.25% Avicel®; FMC BioPolymer, Belgium) was added. Cells were further incubated at 37°C, 5% CO_2 for 24 h. To detect single focus of virus-infected cells, the cells were fixed and permeabilized with 150 μL fixing solution (4% paraformaldehyde; Roth, Germany), 1% Triton X-100 (Roth) in PBS++, and kept at 4°C for 1 h. Thereafter, the solution was discarded and cells were washed 3× with PBS++/0.05% Tween-20 (Roth). Next, the cells were incubated with 50 μL of primary antibody (mouse anti-influenza A virus nucleoprotein mAb, kindly provided by S. Ludwig, Münster, Germany) diluted 1:100 in PBS++ containing 3% bovine serum albumin (BSA; PAN Biotech,

Germany) for 1 h incubation at room temperature (rt). Then, cells were washed 3× with PBS++, 0.05% Tween-20, and incubated again for 1 h at rt with 50 μL secondary antibody (goat antimouse HRP antibody; Santa Cruz Biotechnology) diluted 1:1000 in PBS++ containing 3% BSA. Next, the cells were washed 3× with PBS++ containing 0.05% Tween-20 and incubated with 40 μL AEC (3-amino-9-ethylcarbazole) staining solution (Santa Cruz Biotechnology). After incubation for 40 min at 37°C, the substrate solution was removed and cells were washed 2× with dH_2O to remove salts. Foci were detected and quantified, by scanning of the 96-well plates with a scanner (Epson, Japan) at a resolution of 1200 dpi and analyzed using Photoshop software (Adobe). Results represent means ± standard error of the mean from three independent experiments.

Infection and inhibitor application. Phospholipid scrambalase 1 inhibitor R5421, ethanimidothioic acid: N-[(N-butylthio-N-methylamino)-carbonyloxy]-methyl ester, was purchased from Endotherm (Saarbrücken, Germany). Stock solutions of the inhibitor from 10 mM to 0.01 μM were prepared in dimethyl sulfoxide (DMSO; Roth) and kept at–20°C prior each experiment. A549 and BEAS-2B cells were seeded in 24-well plates at a concentration of 1×10^5 cells per well. Twenty hours later, cells were infected with PR8 virus at an MOI = 1 and incubated with DMEM/BA (for A549) or BEGM/BA (for BEAS-2B cells) medium containing 0.2% BA (Sigma), 0.5 μg mL^{-1} TPCK-treated trypsin, and R5421 inhibitor at different concentrations (0, 0.0001, 0.001, 0.01, 0.1, 1, 10, or 100 μM). As a control, cells treated only with equivalent amount of solvent (DMSO) were used. Twenty-four hours postinfection, supernatants were collected and virus titers were determined by focus forming assay.

Viability test. A549 and BEAS-2b cells were seeded at 96-well plates with a flat bottom. Twenty-four hours later, 90 μL of complete DMEM for A549 cells or BEGM for BEAS-2B cells containing inhibitor R5421 at the end concentration—0.0001, 0.001, 0.01, 0.1, 1, 10, and 100 μM—was added. As a control, DMSO was used. Twenty-four hours post-treatment viability of cells was analyzed by application of PrestoBlue reagent (Thermo Fisher Scientific, Invitrogen) according to the manufactor's protocol. Viability of DMSO-treated cells was set as 100%.

Statistics. Statistical analysis was performed by one-way analysis of variance, Graph Pad Prism 5 Software (GraphPad Software, Inc.).

Supplementary References

S1. Kaufman L, Rousseeuw PJ. Clustering Large Applications (Program CLARA). In: *Finding Groups in Data*. (Kaufman L, Rousseeuw PJ; eds). John Wiley & Sons, Inc., New York, NY, 2008; pp. 126–163.
S2. MacQueen J. Some methods for classification and analysis of multivariate observations. In: *Proceedings of the Fifth Berkeley Symposium on Mathematical Statistics and Probability, Volume 1: Statistics*. University of California Press, Berkeley, CA. 1967; pp. 281–297.
S3. Kaufman L, Rousseeuw PJ. Partitioning Around Medoids (Program PAM). In: *Finding Groups in Data*. (Kaufman L, Rousseeuw PJ; eds). John Wiley & Sons, Inc., New York, NY, 2008; pp. 68–125.
S4. Ernst J, Bar-Joseph Z. STEM: a tool for the analysis of short time series gene expression data. BMC Bioinformatics. 2006;7:191.
S5. Wittkop T, Emig D, Lange S, et al. Partitioning biological data with transitivity clustering. Nat Methods. 2010;7:419–420.
S6. Tan P-N. *Introduction to Data Mining*. Pearson Education India. 2006.
S7. Borgelt C. *Prototype-Based Classification and Clustering*. Otto-von-Guericke-Universität Magdeburg, Universitätsbibliothek, Magdeburg. 2006.
S8. Voyles RM, Morrow JD, Khosla P. Gesture-based programming for robotics: human augmented software adaptation. IEEE Intelligent Systems and their Applications, 1999.
S9. Chaiken D, Foster M. Human-augmented, automatic speech recognition engine. Google Patents US20020152071A1. 2002.
S10. Laczny CC, Sternal T, Plugaru V, et al. VizBin—an application for reference-independent visualization and human-augmented binning of metagenomic data. Microbiome. 2015;3:1.
S11. Ray J, Pinar A, Seshadhri C. A stopping criterion for Markov chains when generating independent random graphs. J Complex Netw. 2015;3: 204–220.
S12. Alcaraz N, Pauling J, Batra R, et al. KeyPathwayMiner 4.0: condition-specific pathway analysis by combining multiple omics studies and networks with Cytoscape. BMC Syst Biol. 2014;8:99.
S13. Ma W, Brenner D, Wang Z, et al. The NS segment of an H5N1 highly pathogenic avian influenza virus (HPAIV) is sufficient to alter replication efficiency, cell tropism, and host range of an H7N1 HPAIV. J Virol. 2010;84:2122–2133.
S14. Malur M, Gale M, Jr., Krug RM. LGP2 downregulates interferon production during infection with seasonal human influenza A viruses that activate interferon regulatory factor 3. J Virol. 2012;86: 10733–10738.
S15. Si-Tahar M, Blanc F, Furio L, et al. Protective role of LGP2 in influenza virus pathogenesis. J Infect Dis. 2014;210:214–223.
S16. Kumar A, Vijayakumar P, Gandhale PN, et al. Genome-wide gene expression pattern underlying differential host response to high or low pathogenic H5N1 avian influenza virus in ducks. Acta Virol. 2017;61: 66–76.
S17. Carter CJ. Schizophrenia susceptibility genes directly implicated in the life cycles of pathogens: cytomegalovirus, influenza, herpes simplex, rubella, and Toxoplasma gondii. Schizophr Bull. 2009;35: 1163–1182.
S18. Zhu Z, Shi Z, Yan W, et al. Nonstructural protein 1 of influenza A virus interacts with human guanylate-binding protein 1 to antagonize antiviral activity. PLoS One. 2013;8:e55920.
S19. Zhu Z, Wei J, Shi Z, et al. Identification of human guanylate-binding protein 1 gene (hGBP1) as a direct transcriptional target gene of p53. Biochem Biophys Res Commun. 2013;436:204–211.
S20. Tang Y, Zhong G, Zhu L, et al. Herc5 attenuates influenza A virus by catalyzing ISGylation of viral NS1 protein. J Immunol. 2010;184:5777–5790.
S21. Dastur A, Beaudenon S, Kelley M, et al. Herc5, an interferon-induced HECT E3 enzyme, is required for conjugation of ISG15 in human cells. J Biol Chem. 2006;281:4334–4338.
S22. Neumann G, Noda T, Kawaoka Y. Emergence and pandemic potential of swine-origin H1N1 influenza virus. Nature. 2009;459:931–939.

S23. Kobasa D, Jones SM, Shinya K, et al. Aberrant innate immune response in lethal infection of macaques with the 1918 influenza virus. Nature. 2007;445:319–323.
S24. Pichlmair A, Lassnig C, Eberle CA, et al. IFIT1 is an antiviral protein that recognizes 5′-triphosphate RNA. Nat Immunol. 2011;12:624–630.
S25. Diamond MS, Farzan M. The broad-spectrum antiviral functions of IFIT and IFITM proteins. Nat Rev Immunol. 2013;13:46–57.
S26. Iwasaki A, Pillai PS. Innate immunity to influenza virus infection. Nat Rev Immunol. 2014;14:315–328.
S27. Shapira SD, Gat-Viks I, Shum BO, et al. A physical and regulatory map of host-influenza interactions reveals pathways in H1N1 infection. Cell. 2009;139:1255–1267.
S28. Ciancanelli MJ, Huang SX, Luthra P, et al. Infectious disease. Life-threatening influenza and impaired interferon amplification in human IRF7 deficiency. Science. 2015;348:448–453.
S29. Kim TK, Bheda-Malge A, Lin Y, et al. A systems approach to understanding human rhinovirus and influenza virus infection. Virology. 2015; 486:146–157.
S30. Morales DJ, Monte K, Sun L, et al. Novel mode of ISG15-mediated protection against influenza A virus and Sendai virus in mice. J Virol. 2015;89:337–349.
S31. Espert L, Degols G, Gongora C, et al. ISG20, a new interferon-induced RNase specific for single-stranded RNA, defines an alternative antiviral pathway against RNA genomic viruses. J Biol Chem. 2003;278:16151–16158.
S32. Degols G, Eldin P, Mechti N. ISG20, an actor of the innate immune response. Biochimie. 2007;89:831–835.
S33. Wilk E, Pandey AK, Leist SR, et al. RNAseq expression analysis of resistant and susceptible mice after influenza A virus infection identifies novel genes associated with virus replication and important for host resistance to infection. BMC Genomics. 2015;16:655.
S34. Tumpey TM, Szretter KJ, Van Hoeven N, et al. The Mx1 gene protects mice against the pandemic 1918 and highly lethal human H5N1 influenza viruses. J Virol. 2007;81:10818–10821.
S35. Shin DL, Hatesuer B, Bergmann S, et al. Protection from severe influenza virus infections in mice carrying the Mx1 influenza virus resistance gene strongly depends on genetic background. J Virol. 2015;89: 9998–10009.
S36. Seo SU, Kwon HJ, Song JH, et al. MyD88 signaling is indispensable for primary influenza A virus infection but dispensable for secondary infection. J Virol. 2010;84:12713–12722.
S37. Ranjan P, Singh N, Kumar A, et al. NLRC5 interacts with RIG-I to induce a robust antiviral response against influenza virus infection. Eur J Immunol. 2015;45:758–772.
S38. Talukder AH, Bao M, Kim TW, et al. Phospholipid scramblase 1 regulates Toll-like receptor 9-mediated type I interferon production in plasmacytoid dendritic cells. Cell Res. 2012;22:1129–1139.
S39. Iki S, Yokota S, Okabayashi T, et al. Serum-dependent expression of promyelocytic leukemia protein suppresses propagation of influenza virus. Virology. 2005;343:106–115.
S40. Gupta G, Pardington P, Chaudhary A, et al. Host innate immune responses are different for high and low pathogenicity influenza A virus subtypes (INM8P.443). J. Immunol. 2014;192:124.129-124.129.
S41. Chelbi-Alix MK, Quignon F, Pelicano L, et al. Resistance to virus infection conferred by the interferon-induced promyelocytic leukemia protein. J. Virol. 1998;72:1043–1051.
S42. García-Sastre A, Durbin RK, Zheng H, et al. The role of interferon in influenza virus tissue tropism. J. Virol. 1998;72:8550–8558.
S43. Ramirez IA, Caverly LJ, Kalikin LM, et al. Differential responses to rhinovirus- and influenza-associated pulmonary exacerbations in patients with cystic fibrosis. Ann Am Thorac Soc. 2014;11:554–561.
S44. Tyner JW, Uchida O, Kajiwara N, et al. CCL5-CCR5 interaction provides antiapoptotic signals for macrophage survival during viral infection. Nat Med. 2005;11:1180–1187.
S45. Culley FJ, Pennycook AM, Tregoning JS, et al. Role of CCL5 (RANTES) in viral lung disease. J Virol. 2006;80:8151–8157.
S46. Le Goffic R, Pothlichet J, Vitour D, et al. Cutting Edge: Influenza A virus activates TLR3-dependent inflammatory and RIG-I-dependent antiviral responses in human lung epithelial cells. J Immunol. 2007; 178:3368–3372.
S47. Jin S, Li Y, Pan R, et al. Characterizing and controlling the inflammatory network during influenza A virus infection. Sci Rep. 2014;4:3799.
S48. Li Y, Li C, Xue P, et al. ISG56 is a negative-feedback regulator of virus-triggered signaling and cellular antiviral response. Proc Natl Acad Sci U S A 2009;106:7945–7950.
S49. Tate MD, Ong JD, Dowling JK, et al. Reassessing the role of the NLRP3 inflammasome during pathogenic influenza A virus infection via temporal inhibition. Sci Rep. 2016;6:27912.
S50. Sarvestani ST, McAuley JL. The role of the NLRP3 inflammasome in regulation of antiviral responses to influenza A virus infection. Antiviral Res. 2017;148:32–42.
S51. Muhlbauer D, Dzieciolowski J, Hardt M, et al. Influenza virus-induced caspase-dependent enlargement of nuclear pores promotes nuclear export of viral ribonucleoprotein complexes. J Virol. 2015;89:6009–6021.
S52. Falschlehner C, Schaefer U, Walczak H. Following TRAIL's path in the immune system. Immunology. 2009;127:145–154.
S53. Danesh A, Seneviratne C, Cameron CM, et al. Cloning, expression and characterization of ferret CXCL10. Mol Immunol. 2008;45:1288–1297.
S54. Jacobs JL, Coyne CB. Mechanisms of MAVS regulation at the mitochondrial membrane. J Mol Biol. 2013;425:5009–5019.
S55. Reikine S, Nguyen JB, Modis Y. Pattern recognition and signaling mechanisms of RIG-I and MDA5. Front Immunol. 2014;5:342.
S56. Zhao C, Hsiang TY, Kuo RL, et al. ISG15 conjugation system targets the viral NS1 protein in influenza A virus-infected cells. Proc Natl Acad Sci U S A 2010;107:2253–2258.
S57. Othumpangat S, Noti JD, McMillen CM, et al. ICAM-1 regulates the survival of influenza virus in lung epithelial cells during the early stages of infection. Virology. 2016;487:85–94.
S58. Marazzi I, Ho JS, Kim J, et al. Suppression of the antiviral response by an influenza histone mimic. Nature. 2012;483:428–433.
S59. Pommerenke C, Wilk E, Srivastava B, et al. Global transcriptome analysis in influenza-infected mouse lungs reveals the kinetics of innate and adaptive host immune responses. PLoS One. 2012;7:e41169.
S60. Zhou X, Michal JJ, Zhang L, et al. Interferon induced IFIT family genes in host antiviral defense. Int J Biol Sci. 2013;9:200–208.
S61. Liu XY, Chen W, Wei B, et al. IFN-induced TPR protein IFIT3 potentiates antiviral signaling by bridging MAVS and TBK1. J Immunol. 2011; 187:2559–2568.
S62. Daffis S, Szretter KJ, Schriewer J, et al. 2′-O methylation of the viral mRNA cap evades host restriction by IFIT family members. Nature. 2010;468:452–456.
S63. Killip MJ, Fodor E, Randall RE. Influenza virus activation of the interferon system. Virus Res. 2015;209:11–22.
S64. Kroger A, Koster M, Schroeder K, et al. Activities of IRF-1. J Interferon Cytokine Res. 2002;22:5–14.
S65. Yim JH, Ro SH, Lowney JK, et al. The role of interferon regulatory factor-1 and interferon regulatory factor-2 in IFN-gamma growth inhibition of human breast carcinoma cell lines. J Interferon Cytokine Res. 2003;23:501–511.
S66. Honda K, Yanai H, Negishi H, et al. IRF-7 is the master regulator of type-I interferon-dependent immune responses. Nature. 2005;434:772–777.
S67. Au-Yeung N, Mandhana R, Horvath CM. Transcriptional regulation by STAT1 and STAT2 in the interferon JAK-STAT pathway. JAKSTAT. 2013;2: e23931.
S68. Jeon YJ, Yoo HM, Chung CH. ISG15 and immune diseases. Biochim Biophys Acta. 2010;1802:485–496.
S69. Zhu Z, Zhang X, Wang G, et al. The laboratory of genetics and physiology 2: emerging insights into the controversial functions of this RIG-I-like receptor. Biomed Res Int. 2014;2014:960190.
S70. Verhelst J, Parthoens E, Schepens B, et al. Interferon-inducible protein Mx1 inhibits influenza virus by interfering with functional viral ribonucleoprotein complex assembly. J Virol. 2012;86:13445–13455.
S71. Kawai T, Sato S, Ishii KJ, et al. Interferon-alpha induction through Toll-like receptors involves a direct interaction of IRF7 with MyD88 and TRAF6. Nat Immunol. 2004;5:1061–1068.
S72. Loo YM, Gale M, Jr. Immune signaling by RIG-I-like receptors. Immunity. 2011;34:680–692.
S73. Ibsen MS, Gad HH, Thavachelvam K, et al. The 2′-5′-oligoadenylate synthetase 3 enzyme potently synthesizes the 2′-5′-oligoadenylates required for RNase L activation. J Virol. 2014;88:14222–14231.
S74. Castelli JC, Hassel BA, Wood KA, et al. A study of the interferon antiviral mechanism: apoptosis activation by the 2–5A system. J Exp Med. 1997;186:967–972.

S75. Takizawa T, Ohashi K, Nakanishi Y. Possible involvement of double-stranded RNA-activated protein kinase in cell death by influenza virus infection. J Virol. 1996;70:8128–8132.
S76. Zhou A, Paranjape JM, Hassel BA, et al. Impact of RNase L overexpression on viral and cellular growth and death. J Interferon Cytokine Res. 1998;18:953–961.
S77. Schierhorn KL, Jolmes F, Bespalowa J, et al. Influenza A virus virulence depends on two amino acids in the N-terminal domain of its NS1 protein to facilitate inhibition of the RNA-dependent protein kinase PKR. J Virol. 2017;91:pii: e00198-17.
S78. Sadler AJ, Williams BR. Structure and function of the protein kinase R. Curr Top Microbiol Immunol. 2007;316:253–292.
S79. Gaur P, Munjhal A, Lal SK. Influenza virus and cell signaling pathways. Med Sci Monit. 2011;17:RA148–RA154.
S80. Oeckinghaus A, Ghosh S. The NF-kappaB family of transcription factors and its regulation. Cold Spring Harb Perspect Biol. 2009;1: a000034.
S81. Krieghoff-Henning E, Hofmann TG. Role of nuclear bodies in apoptosis signalling. Biochim Biophys Acta. 2008;1783:2185–2194.
S82. Zhang M, Tian Y, Wang RP, et al. Negative feedback regulation of cellular antiviral signaling by RBCK1-mediated degradation of IRF3. Cell Res. 2008;18:1096–1104.
S83. Geoffroy MC, Chelbi-Alix MK. Role of promyelocytic leukemia protein in host antiviral defense. J Interferon Cytokine Res. 2011;31:145–158.
S84. Li H, Gade P, Xiao W, et al. The interferon signaling network and transcription factor C/EBP-beta. Cell Mol Immunol. 2007;4:407–418.
S85. Tau G, Rothman P. Biologic functions of the IFN-gamma receptors. Allergy. 1999;54:1233–1251.
S86. Xia Z, Xu G, Yang X, et al. Inducible TAP1 negatively regulates the antiviral innate immune response by targeting the TAK1 complex. J Immunol. 2017;198:3690–3704.
S87. Siednienko J, Gajanayake T, Fitzgerald KA, et al. Absence of MyD88 results in enhanced TLR3-dependent phosphorylation of IRF3 and increased IFN-beta and RANTES production. J Immunol. 2011;186: 2514–2522.
S88. Ishikawa E, Nakazawa M, Yoshinari M, et al. Role of tumor necrosis factor-related apoptosis-inducing ligand in immune response to influenza virus infection in mice. J Virol. 2005;79:7658–7663.
S89. Foss S, Watkinson R, Sandlie I, et al. TRIM21: a cytosolic Fc receptor with broad antibody isotype specificity. Immunol Rev. 2015;268:328–339.
S90. Yang K, Shi HX, Liu XY, et al. TRIM21 is essential to sustain IFN regulatory factor 3 activation during antiviral response. J Immunol. 2009;182:3782–3792.
S91. Di Pietro A, Kajaste-Rudnitski A, Oteiza A, et al. TRIM22 inhibits influenza A virus infection by targeting the viral nucleoprotein for degradation. J Virol. 2013;87:4523–4533.
S92. Fitzgerald KA. The interferon inducible gene: viperin. J Interferon Cytokine Res. 2011;31:131–135.
